# Interdisciplinary approach to the demography of Jamaica

**DOI:** 10.1186/1471-2148-12-24

**Published:** 2012-02-23

**Authors:** Michael L Deason, Antonio Salas, Simon P Newman, Vincent A Macaulay, Errol Y st A Morrison, Yannis P Pitsiladis

**Affiliations:** 1 Institute of Cardiovascular and Medical Sciences, University of Glasgow, Glasgow, UK; 2 Unidade de Xenética, Departamento de Anatomía Patolóxica e Ciencias Forenses, and Instituto de Medicina Legal, Facultade de Medicina, Universidad de Santiago de Compostela, Santiago de Compostela, Galicia, Spain; 3 Department of History, University of Glasgow, Glasgow, UK; 4 Department of Statistics, University of Glasgow, Glasgow, UK; 5 Department of Basic Medical Sciences, University of the West Indies, Kingston, Jamaica

## Abstract

**Background:**

The trans-Atlantic slave trade dramatically changed the demographic makeup of the New World, with varying regions of the African coast exploited differently over roughly a 400 year period. When compared to the discrete mitochondrial haplotype distribution of historically appropriate source populations, the unique distribution within a specific source population can prove insightful in estimating the contribution of each population. Here, we analyzed the first hypervariable region of mitochondrial DNA in a sample from the Caribbean island of Jamaica and compared it to aggregated populations in Africa divided according to historiographically defined segments of the continent's coastline. The results from these admixture procedures were then compared to the wealth of historic knowledge surrounding the disembarkation of Africans on the island.

**Results:**

In line with previous findings, the matriline of Jamaica is almost entirely of West African descent. Results from the admixture analyses suggest modern Jamaicans share a closer affinity with groups from the Gold Coast and Bight of Benin despite high mortality, low fecundity, and waning regional importation. The slaves from the Bight of Biafra and West-central Africa were imported in great numbers; however, the results suggest a deficit in expected maternal contribution from those regions.

**Conclusions:**

When considering the demographic pressures imposed by chattel slavery on Jamaica during the slave era, the results seem incongruous. Ethnolinguistic and ethnographic evidence, however, may explain the apparent non-random levels of genetic perseverance. The application of genetics may prove useful in answering difficult demographic questions left by historically voiceless groups.

## Background

The African Diaspora in the New World provides the unique opportunity to understand the demographic stresses imposed on those forcibly relocated during the trans-Atlantic slave trade. Of the estimated ten million people captured in Africa between the 16^th ^and 19^th ^centuries by European powers, just fewer than nine million survived the harrowing Middle Passage across the Atlantic [1]. Detailed historical records and accounts have been synthesized over the past 50 years, allowing for valuable demographic reconstructions of various populations by period and particular region of origin. The vast majority of Africans arriving in the British America were enslaved as plantation labourers--a result of growing economic demand in Europe for agricultural luxuries such as sugar and tobacco. By the abolition of slave importation in the British Empire in 1807, roughly 2.6 million people had been uprooted and relocated to British America [1].

The island of Jamaica was sparsely inhabited by indigenous sea faring peoples when it was established as a Spanish settlement in 1509. These peoples either fled the island or were eradicated by the time of the English conquest of Jamaica in 1655, the result of forced labour and European diseases imposed by the Spanish [2]. Due to its lack of precious metals, the Spanish sparsely populated the island and only minimally invested in light agriculture and livestock. In 1612 and 1613, the only documented African slaves arrived on the island under the Spanish crown--503 in total [1]. The English, however, were quick to establish intensive slave labour sugar plantations similar to those already proving profitable on the nearby British colony of Barbados. The economy of Jamaica diversified over time, with coffee and pimento eventually joining sugar as agricultural exports [3]. An estimated 927,000 slaves disembarked from Africa on the island between 1655 and the successful abolition of the slave trade in 1807 [1]; however, in an estimate of slave numbers made in 1808, the population only numbered 354,000. By the abolition of chattel slavery in 1834, the population dropped further to 311,070 [3]. The result of a number of demographic and health related scenarios, the obvious disparities in these values indicate an environment not conducive to reproduction with replacement in the slave population of Jamaica.

**Figure 1:**
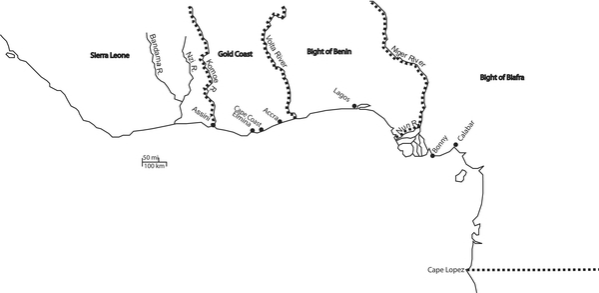
**Africa in the era of the trans-Atlantic slave trade**. Map used by permission from Christelle Le Riguer.

Between 1655 and 1807, captive Africans embarked from the most westerly shores of Senegambia and along the coast eastward, as far as Madagascar. These regions (Figure 1) were not represented uniformly between 1655 and 1807 in Jamaica, illustrated in Table 1. This variability resulted from a number of multifaceted economic pressures. The arrival of trade with Europe introduced a continental outlet for an already common practice; slavery was widespread in Africa largely because people were the only form of private, revenue-producing property recognized across the continent [4]. Highly regulatory European trading companies established forts and slave factories along the West African coast in places like the Gold Coast and Bight of Benin which increasingly led to civil unrest among participatory African nations, a consequence that led to the further enslavement of conquered groups. While most groups would sell prisoners acquired during conflicts, some populations went as far as undertaking specialized military campaigns in order to procure more slaves for the Atlantic trade [5]. Inter-African political variability also led to changes in slave exportation. The most developed areas of Africa at the time of European contact were also those most likely to be drawn into the trans-Atlantic slave trade. Only the most politically unified and densely populated areas could foster the early commodity trade with Europe; when European interest turned to human cargo, these areas were most directly affected by the slave trade [6]. These more densely populated areas had the capacity to respond to the explosive increase in demand during the middle and late 18^th ^century.

**Table 1:** Imputed number of slave disembarked in Jamaica between 1655 and 1808 for voyages with known embarkation points [1]

	Senegambia	Sierra Leone	Gold Coast	Bight of Benin	Bight of Biafra	West-central Africa	Southeast Africa	Total
1651-1660	-	-	-	-	85	-	-	85
1661-1670	-	-	-	704	2,101	500	-	3,305
1671-1680	158	-	2,402	2,071	2,204	1,211	462	8,508
1681-1690	960	92	412	9,465	2,445	7,139	171	20,684
1691-1700	1,447	205	1,270	3,181	1,926	3,593	-	11,622
1701-1710	1,328	413	13,445	8,918	730	2,843	-	27,677
1711-1720	813	125	12,319	6,636	324	1,497	385	22,099
1721-1730	1,603	667	12,055	7,761	3,011	6,003	-	31,100
1731-1740	303	559	6,387	954	4,795	7,708	-	20,706
1741-1750	1,993	2,142	10,062	1,155	14,868	7,134	-	37,354
1751-1760	2,049	8,591	22,630	5,032	18,277	10,803	-	67,382
1761-1770	634	11,523	24,387	9,264	18,347	5,133	-	69,288
1771-1780	2,021	11,874	25,980	8,798	25,051	4,282	-	78,006
1781-1790	1,135	5,030	36,289	7,617	34,800	3,546	-	88,417
1791-1800	828	12,322	20,091	5,773	68,391	46,382	-	153,787
1801-1810	246	3,260	12,763	2,882	29,248	12,970	-	61,369

**Total**	**15,518**	**56,803**	**200,492**	**80,211**	**226,603**	**120,744**	**1,018**	**701,389**

Invaluable to modern genealogical research, the mitochondrial genome (mtDNA) is passed entirely matrilineally and accumulates mutations along the maternal line, providing a unique opportunity to explore the matriline of select groups. Once considering the relatively rapid mutation rate of mtDNA when compared to the nuclear genome, it is possible to create detailed phylogenies to explore the matrilineal relatedness of people [7]. Grouping particular haplotypes into larger monophyletic clades (haplogroups) creates easily comparable units of genealogical information; when found in other parts of the world, these haplogroups can be used as indicators of recent migration. Haplogroup frequency distributions are also sensitive to population history: the haploid nature of the mtDNA reduces effective population size and so enhances the effect of genetic drift. The African contribution to the New World matrilineal genealogies in people for recent African origin are far from homozygous [8]; each group of people has its own distinct haplogroup profile distribution shaped by not only point of embarkation and number of founders, but also gross environmental and life history constraints. Earlier mtDNA assessments of the island of Jamaica results suggest an almost entirely West African matrilineal origin to the modern Jamaican population with very few genetic inroads by either Eurasian or Asian/New World matrilines [9,10], consistent with the historical accounts of not only a high slave importation rate, but also a very small resident European female population size [11], and the decimation of indigenous groups almost directly after contact [2].

Using historically defined geographical parameters, the distribution of mtDNA haplogroup profiles in discrete founding populations can be combined in hopes of identifying and replicating the source populations found on Jamaica during the slave trade era. This approach has been used with success in comparing great swaths of New World populations of African origin to various macro-regions in Africa, showing a concurrence with the historical literature, as well as differing contributions of African source populations in North, Central, and South America [8]. Using an expanded and more comprehensive set of source populations, a follow-up study [12] highlighting African-Americans was able to show a roughly 55% similarity in distribution of that group with modern West Africa. Recent reassessments of New World populations of African descent highlight the effect embarkation points and colonial networks had on demographic make-up [13]. Additionally, a similar approach was applied to the Noir Marron of French Guiana, showing the greatest genetic similarity with populations from the Bight of Benin, despite origins ranging from the Ivory Coast to Angola [14].

The aim of this study is to apply similar admixture modelling to the mtDNA distribution of Jamaica with an eye toward geographic sensitivity, using historical data on the coasts of origin as source populations in an attempt to investigate the genetic vestiges of population constraints present during the slave era in Jamaica. Considering the overwhelming proportion of slaves imported from the Bight of Biafra and West-central Africa just before the end of the slave trade, as well as the continuously high levels of mortality among slaves, it is hypothesized that the mtDNA haplogroup profile distribution will resemble these latter sources more closely than regions exploited earlier in the slave trade.

## Results

### Jamaican haplogroups

The mitochondrial haplogroup profiles observed in the sample of Jamaicans are presented in Additional file 1: Table S1. Individual haplotypes were clustered into larger haplogroups to facilitate meaningful comparisons between the groups and facilitate correspondence with the literature, presented in Additional file 2: Table S2. The great majority of the profiles observed in Jamaica could be allocated to L sub-Saharan haplogroups (97.5%), a result echoing past studies showing very few non-African maternal lineages in Jamaica [9]. Two "other L" categories ("Other L0'1'2" and "Other L3") were included for haplogroup sub groupings occurring at low frequencies in the Jamaican sample e.g. L2d and L3h. Combined, the non-Sub-Saharan paragroup accounted for 2.5% of the typed Jamaicans. The North African haplogroup U6 was included separately given its arrival to the Sub-Saharan region as the result of migration prior to the Atlantic slave trade [15]. An additional paragroup (non-L/U6) was included for all haplogroups not commonly found in Sub-Saharan Africa. A total of ten sampled Jamaicans were haplogrouped as non-Sub-Saharan: One being typed haplogroup A2, one B2, and another two D4, all Asian/New World haplogroups. The remaining non-Sub-Saharan haplogroups included one H, two J, and one U2, all Eurasian haplogroups. Two individual were also typed to worldwide haplogroup M.

Only one Jamaican profile could be undoubtedly classified as belonging to typical Native American haplogroups A2; several exact matches were observed in El Salvador [16], Costa Rica [17], and in the 'Hispanic' US sample of [18], supporting its Central American nature and perhaps either one of the few vestiges of the Native American past of the Caribbean before the arrival of trans-Atlantic colonizers and slaves. Another haplotype most likely belong to the Native American haplogroup B2, but the resolution of the HVS-I alone only allows its classification as belonging to haplogroup B4. There are several lineages most likely belonging to different sub-clades of the Pan-Asian haplogroup D4, and a member of haplogroup M30c1 of presumable Indian origin. Haplogroup U6 is typically from North Africa; however, the U6a profile observed in Jamaica (T16172C C16184T A16219G C16234T C16278T T16311C) has been reported in the Ikot Mbonde from Southeast Nigeria [19] but not in North Africa. On the other hand, there are few haplotypes of European ancestry in Jamaica. The H2a2b1 profile A16235G C16291T A16293G was observed in Bolzano (Italy) [20], in Galicia (Spain) [21]; in the USA, this haplotype is quite common as it appeared many times in the Genographic database [22]. Curiously, no matches have been found for some of the European profiles, e.g. the J haplotype T16126C C16187T T16189C and the U2 haplotype A16051G A16206C C16291T T16359C. The presence of these lineages in the modern population may highlight the rapid assimilation of a small albeit global workforce arriving to a post-emancipation Jamaica during the subsequent labour shortage.

As shown in Additional file 3: Table S3 and Additional file 4: Table S4, the great majority of Jamaican haplotypes are present in populations sampled along the coastline of West Africa. Only eight different Jamaican haplotypes have frequencies above 2%. Of these, three of them are more frequent along the Guinea Coast than in any other African regions, while the remaining five are often found in the entirety of sub-Saharan Africa. The L2a1 profile C16223T C16278T C16294T A16309G G16319A has been found nine times in Jamaica, more than any other African or American region, indicating the existence of discrete genetic drift episodes on the island. Typical East African haplogroups, such as L4 and L5 profiles are virtually absent in Jamaica. There is only one member belonging to L4b1which is, however, one of the two common sub-lineages of L4 in the Bight of Biafra and the Gold Coast. This is in agreement with the historical documentation indicating that East Africa was not targeted by the European naval powers for slave trading across the Atlantic.

### Admixture analyses

The results of the exact test of population differentiation performed on the haplogroup profile distributions confirm the discrete nature of each African coast (results not shown). In order to estimate the proportion of maternal ancestry from each major slaving coast present in the population of modern Jamaican mtDNA, admixture models using both haplogroup profiles and haplotype similarities from the African coasts were fitted to the pool of sampled Jamaicans. Combinations excluding marginal populations were also explored to investigate any contribution these groups may have on the regional haplogroup profile distribution. These estimated admixture coefficients are summarized in Tables 2 and 3, respectively.

**Table 2:** Admixture coefficients ± SD for parental populations calculated using haplogroup profile distributions

All	Jamaica (n = 390)
Senegambia (n = 892)	0.049 ± 0.040
Sierra Leone (n = 823)	0.096 ± 0.07
Gold Coast (n = 505)	0.477 ± 0.12
Bight of Benin (n = 421)	0.123 ± 0.10
Bight of Biafra (n = 3641)	0.064 ± 0.05
West-central Africa (n = 1403)	0.089 ± 0.05
Southeast Africa (n = 775)	0.092 ± 0.03
East Africa (n = 805)	0.010 ± 0.01
**w/o Sahelian**	**Jamaica (n = 390)**

Senegambia (n = 39)	0.075 ± 0.05
Sierra Leone (n = 659)	0.092 ± 0.07
Gold Coast (n = 491)	0.336 ± 0.15
Bight of Benin (n = 297)	0.261 ± 0.15
Bight of Biafra (n = 3008)	0.060 ± 0.05
West-central Africa (n = 1403)	0.081 ± 0.05
Southeast Africa (n = 775)	0.088 ± 0.03
East Africa (n = 805)	0.009 ± 0.01
**w/o Pygmies**	**Jamaica (n = 390)**

Senegambia (n = 892)	0.048 ± 0.04
Sierra Leone (n = 823)	0.092 ± 0.07
Gold Coast (n = 505)	0.456 ± 0.11
Bight of Benin (n = 421)	0.105 ± 0.09
Bight of Biafra (n = 3097)	0.095 ± 0.08
West-central Africa (n = 1314)	0.109 ± 0.06
Southeast Africa (n = 775)	0.085 ± 0.03
East Africa (n = 753)	0.009 ± 0.009
**w/o Sahelian or Pygmies**	**Jamaica (n = 390)**

Senegambia (n = 39)	0.072 ± 0.04
Sierra Leone (n = 659)	0.092 ± 0.07
Gold Coast (n = 491)	0.343 ± 0.14
Bight of Benin (n = 297)	0.214 ± 0.14
Bight of Biafra (n = 2464)	0.091 ± 0.07
West-central Africa (n = 1314)	0.097 ± 0.06
Southeast Africa (n = 775)	0.083 ± 0.03
East Africa (n = 753)	0.008 ± 0.01

**Table 3:** Ancestry proportions based on a Bayesian approach on haplotype frequencies; *n*: sample size.

	Jamaica					
All	*P*(0)	*95% CI*	*P*(1)	*95% CI*	*P*(2)	*95% CI*
Senegambia (*n *= 892)	0.1159	0.1096-0.1222	0.1239	0.1175-0.1304	0.1399	0.1331-0.1467

Sierra Leone (*n *= 823)	0.1431	0.1362-0.1500	0.1453	0.1384-0.1522	0.1479	0.1409-0.1548

Gold Coast (*n *= 505)	0.1956	0.1878-0.2033	0.1724	0.1650-0.1798	0.1642	0.1570-0.1715

Bight of Benin (*n *= 400)	0.1704	0.1630-0.1778	0.1745	0.1671-0.1819	0.1623	0.1550-0.1695

Bight of Biafra (*n *= 3641)	0.1501	0.1431-0.1571	0.1408	0.1340-0.1476	0.1335	0.1268-0.1402

West-central Africa (*n *= 1403)	0.1099	0.1037-0.1160	0.1116	0.1054-0.1178	0.1124	0.1062-0.1186

South East Africa (*n *= 775)	0.0580	0.0534-0.0626	0.0768	0.0716-0.0820	0.0865	0.0810-0.0920

East Africa (*n *= 805)	0.0571	0.0525-0.0616	0.0547	0.0503-0.0592	0.0533	0.0489-0.0577

	**Jamaica**					
**w/o Sahelian**	*P*(0)	*95% CI*	*P*(1)	*95% CI*	*P*(2)	*95% CI*

Senegambia (*n *= 39)	0.1046	0.0986-0.1106	0.0977	0.0919-0.1035	0.1215	0.1151-0.1279

Sierra Leone (*n *= 659)	0.1397	0.1329-0.1465	0.1544	0.1473-0.1615	0.1576	0.1505-0.1648

Gold Coast (*n *= 491)	0.1948	0.1871-0.2026	0.1752	0.1677-0.1826	0.1676	0.1603-0.1749

Bight of Benin (*n *= 297)	0.1878	0.1801-0.1954	0.1904	0.1827-0.1980	0.1695	0.1622-0.1769

Bight of Biafra (*n *= 3008)	0.1565	0.1494-0.1636	0.1449	0.1380-0.1518	0.1341	0.1274-0.1408

West-central Africa (*n *= 1403)	0.1071	0.1010-0.1131	0.1081	0.1020-0.1142	0.1103	0.1042-0.1164

South East Africa (*n *= 775)	0.0555	0.0510-0.0600	0.0761	0.0709-0.0813	0.0859	0.0804-0.0914

East Africa (*n *= 805)	0.0540	0.0496-0.0585	0.0533	0.0489-0.0577	0.0534	0.0490-0.0578

	**Jamaica**					
**w/o Pygmies**	***P*(0)**	***95% CI***	***P*(1)**	***95% CI***	***P*(2)**	***95% CI***

Senegambia (*n *= 892)	0.1149	0.1087-0.1212	0.1212	0.1148-0.1276	0.1371	0.1304-0.1438

Sierra Leone (*n *= 823)	0.1391	0.1324-0.1459	0.1404	0.1336-0.1472	0.1436	0.1368-0.1505

Gold Coast (*n *= 505)	0.1913	0.1836-0.1990	0.1712	0.1638-0.1785	0.1613	0.1541-0.1685

Bight of Benin (*n *= 421)	0.1668	0.1595-0.1741	0.1690	0.1616-0.1763	0.1575	0.1503-0.1646

Bight of Biafra (*n *= 3097)	0.1648	0.1575-0.1721	0.1543	0.1472-0.1614	0.1455	0.1386-0.1525

West-central Africa (*n *= 1314)	0.1112	0.1050-0.1174	0.1137	0.1075-0.1199	0.1162	0.1099-0.1224

South East Africa (*n *= 775)	0.0562	0.0517-0.0608	0.0755	0.0703-0.0806	0.0851	0.0797-0.0906

East Africa (*n *= 753)	0.0556	0.0511-0.0601	0.0548	0.0503-0.0592	0.0537	0.0493-0.0581

	**Jamaica**					
**w/o Sahelians or Pygmies**	***P*(0)**	***95% CI***	***P*(1)**	***95% CI***	***P*(2)**	***95% CI***

Senegambia (*n *= 39)	0.1012	0.0953-0.1071	0.0945	0.0888-0.1002	0.1179	0.1116-0.1242

Sierra Leone (*n *= 659)	0.1348	0.1281-0.1415	0.1484	0.1415-0.1554	0.1523	0.1452-0.1593

Gold Coast (*n *= 491)	0.1891	0.1814-0.1967	0.1710	0.1637-0.1784	0.1632	0.1559-0.1704

Bight of Benin (*n *= 297)	0.1803	0.1728-0.1878	0.1831	0.1756-0.1907	0.1636	0.1563-0.1708

Bight of Biafra (*n *= 2463)	0.1735	0.1661-0.1809	0.1609	0.1537-0.1681	0.1483	0.1414-0.1553

West-central Africa (*n *= 1314)	0.1096	0.1035-0.1157	0.1106	0.1045-0.1168	0.1149	0.1086-0.1211

South East Africa (*n *= 775)	0.0554	0.0509-0.0599	0.0753	0.0701-0.0804	0.0841	0.0786-0.0895

East Africa (*n *= 753)	0.0561	0.0516-0.0606	0.0561	0.0516-0.0606	0.0558	0.0513-0.0603

*P*(x) refers to the freedom in haplotype matching, e.g. *P*(0) are perfect matches and *P*(2) are 2 differences from the shared haplotype

Varying parts of the western coast of Africa contributed to the British trans-Atlantic slave trade in differing intensities over differing periods; however, the Gold Coast embarked slaves for the New World in consistently great numbers between the beginning of the 18^th ^century and the 1790 s.

Using haplogroup distributions to calculate parental population contribution, the largest admixture coefficient was associated with the Gold Coast (0.477 ± 0.12), suggesting that the people from this region may have been consistently prolific throughout the slave era on Jamaica. The diminutive admixture coefficients associated with the Bight of Biafra and West-central Africa (0.064 ± 0.05 and 0.089 ± 0.05, respectively) is striking considering the massive influx of individuals from these areas in the waning years of the British Slave trade. When excluding the pygmy groups, the contribution from the Bight of Biafra and West-central rise to their highest levels (0.095 ± 0.08 and 0.109 ± 0.06, respectively), though still far from a major contribution. When admixture coefficients were calculated by assessing shared haplotypes, the Gold Coast also had the largest contribution, though much less striking at 0.196, with a 95% confidence interval of 0.189 to 0.203. Interestingly, when haplotypes are allowed to differ by one base pair, the Jamaican matriline shows the greatest affinity with the Bight of Benin, though both Bight of Biafra and West-central Africa remain underrepresented.

### Diversity and demographic analyses

The results of the evaluation on genetic diversity and demography of the parental populations and of the Jamaican mitochondrial gene pool are summarized in Table 4. All parental groups show expectedly high levels of diversity with regards to θπ, as well as signs of expansion with regards to the unimodal stepwise expansion indices RI and SSD. All groups produced negative values for Tajima's D, also indicating population expansion and departure from the mutation drift equilibrium, although West-central and Southeast Africa failed to produce significant p-values. Sampled Jamaicans fall within the range for diversity and demographic indices, providing no evidence of founder effect.

**Table 4:** Genetic diversity and demographic statistics for the Jamaicans of African maternal decent and comparative population samples

Sample	n	k	S	θπ ± SD	D	RI	SSD
Jamaica	390	231	95	7.28 ± 3.42	-1.46*	0.0044	0.0009
Senegambia	892	361	118	6.93 ± 3.26	-1.60**	0.0037	0.0010
Sierra Leone	823	343	110	7.21 ± 3.38	-1.48*	0.0035	0.0016
Gold Coast	505	223	96	6.64 ± 3.14	-1.53*	0.0045	0.0007
Bight of Benin	421	196	97	6.75 ± 3.18	-1.58**	0.0048	0.0009
Bight of Biafra	3641	852	149	8.90 ± 4.10	-1.27*	0.0024	0.0014
West-central Africa	1403	370	122	9.05 ± 4.17	-1.15	0.0036	0.0004
South-east Africa	775	228	103	8.50 ± 3.94	-1.14	0.0038	0.0008
East Africa	805	402	139	8.76 ± 4.05	-1.55*	0.0043	0.0005

* *p*-value < 0.05

** *p*-value < 0.02

k = number of distinct haplotypes

S = number of polymorphic sites

θπ ± SD = mean number of pairwise differences

D = Tajima's D

*RI *raggedness index

*SSD *sum of square deviations

## Discussion

The African Diaspora in Jamaica is the result of a well-documented trade in human lives for just over 150 years motivated almost entirely by the rise in demand for luxury goods in Western Europe. By taking historical African embarkation points into account, we compared estimates of maternal contribution of each parental population with historical disembarkation records. The results of the admixture analysis suggest the mtDNA haplogroup profile distribution of Jamaica more closely resembles that of aggregated populations from the modern day Gold Coast region despite an increasing influx of individuals from both the Bight of Biafra and West-central Africa during the final years of the trade. When taking what is known about the negative rate of natural population growth of slaves on Jamaica, these results add an additional layer of complexity to demographic history of Jamaica. Planters found it more economical to import new labour rather than invest in natural reproduction within their existing groups. Coupling low fecundity with the high mortality leads to the expectation of a fluid demographic shift through time to a haplogroup profile distribution more closely resembling those groups arriving later during the slave trade. Present results do not show this, hinting instead at non-random processes in the creation of modern Jamaican matrilineal demography.

The admixture results may suggest a preference among Jamaican planters. Historic evidence suggests the Jamaican planting class held the Akan of the Gold Coast in very high regards [23], although similar anecdotal evidence is mixed [24]. Individual planters may have had ethnic preferences, although it would have been unwise to ignore immediate labour demands. The Jamaican slave market was typified by large purchasers competing for limited number of Africans [24]. The sale of slaves in Kingston, Jamaica's main slaving port, was characterized by timing and market savvy. Prices varied considerably. Often the most desirable slaves were sold at the beginning of the sale in very small numbers for well over the average price of the total sale [25]. As the sale progressed, prices dropped, and often Kingston merchants would make very large purchases for less than the price as a whole. These slaves would then be transported to urban yards to be acclimatized to Jamaica and slavery with individuals from other shipments, and then resold for a profit to planters [24]. The whole process would have resulted in a more heterogeneous cultural mix even before reaching their final destination.

The entire acclimatization process was understandably both mentally and physically stressful for newly arriving Africans. Between a quarter to a half of newly landed Africans died within the first three years on the island [26]. The distance and time individuals spent travelling is negatively correlated with survival [27]; as such, individuals embarking at ports further from Jamaica would be expected to arrive in a more poorly state. It can postulated therefore that despite more than half of all Africans shipped to Jamaica coming from the Bight of Biafra, they may have not survived the acclimatization process as frequently as those individuals from further west along the coast. Individuals arriving from Southeast Africa and Madagascar were significantly disadvantaged in this respect, perhaps evidenced by their negligible contribution to the mtDNA pool of Jamaica.

The slave society on Jamaica also operated in a very rigid social hierarchy; creole slaves had much greater life expectancy, fecundity, and upward social mobility than those born in Africa [3]. The entire society was also highly endogamous, the one glaring omission being the high frequency in which white men fathered children with their slaves, providing an opportunity for intergenerational mobility. Slaves born of mixed parentage were more often the recipients of more favourable positions, including domestics and tradesmen. Slaves of colour were also much more likely to be manumitted by their owners [28]. Considering the estimated paternal contribution by Europeans for modern Jamaicans is estimated at just over 40% [10], African-European admixture may have played an important role in the legacy of the slave population.

The development of modern Jamaican English may also provide insight into the demographic development of the island. The modern creolized English spoken on the island has been traced to relatively uneducated Northern British and Irish overseers and bookmakers and the early African slaves they interacted with. During the initial era of slavery on the island (1655-1700), slave acculturation was a process characterized by direct contact between newly arrived Africans and their European overseers. Though the Gold Coast contributed marginally to the slave trade prior to 1700, the Akan speaking groups from modern Ghana were thought to be the largest concentrated linguistic groups [29]. These early slaves heavily influenced the development of the Creole slave language and culture on the island [30]. Additionally, modern Jamaican English contains many loanwords of African origin, a majority of those etymologically from Gold Coast region [31]. A large part of the pidginization is thought to have been completed within the first few decades, and as the proportion of Europeans began to shrink with the explosive increase in slave imports, newly arrived Africans would be more reliant on established slaves for the acquisition of a common tongue [32]. Contemporary accounts of a 'two-burial' custom also match those found in groups from the Gold Coast [33]. Africans arriving from the Gold Coast may have thus found the acclimatization and acculturation process less stressful because of cultural and linguistic commonalities, leading ultimately to a greater chance of survivorship and a greater number of progeny.

## Conclusions

In summation, despite the historical evidence that an overwhelming majority of slaves were sent from the Bight of Biafra and West-central Africa near the end of the British slave trade, the mtDNA haplogroup profile of modern Jamaicans show a greater affinity with groups found in the present day Gold Coast region. Caution must be paid however to the scope of the analyses performed here. The Jamaican slave markets were the largest in the West Indies and sporadic accounts exist of slaves being purchase in Jamaica for plantations in other part of the New World; however, it is difficult to accurately trace the ancestry of the resold slaves. Additionally, after the abolition of slavery in 1834, the island is treated here as roughly a closed system with regards to the African continent. The trajectory of the mtDNA distribution is assumed to have stayed relatively consistent since emancipation; however, constraints imposed on the Jamaican population may have changed through time, influencing modern demography. The end of the slave trade in Jamaica brought about a change in economic climate, with a small albeit recognizable amount of Jamaicans emigrating to other parts of the world, as well as foreign migrant labours arriving from around the globe. Whether any these constraints have significantly affected the mtDNA distribution on the island is difficult to say.

## Methods

### Sampling protocol and criteria

The study was approved by the local ethics committees of the University of West Indies in Mona, Jamaica. After providing informed consent, over 400 Jamaican volunteers were then asked to complete a simple questionnaire stating their birth place, their parents' birth places, and--if known--the birth places of both sets of grandparents. Individuals born outside the country or with any reported maternal relatives born outside the country were excluded from the study. Additionally, where more than one maternal relative was sampled, only one individual was used in further analysis.

### Population comparisons and HVS-I database

A database of 9,265 African first hyper variable segment (HVS-1) sequences was amassed from the literature in order to investigate any life history constraints present on the island during and after slavery. African ethnic groups were assigned locations based on present day ranges of the population imposed on the historic guidelines for the differing coasts. To account for transit of slaves from inland Africa to the coast, these regions were then segregated into the hinterland roughly perpendicularly from the coast, an assumption explored previously [6]. When the location of sample collection is not specified in the literature, the ethnic group's present location according to Ethnologue [34] is used. The following eight historic regional definitions are employed for dividing the African coast: Senegambia is anywhere north of the Nuñez River. Sierra Leone comprises the Nuñez River near Boké, Guinea up to and including the Assini River in Côte d'Ivoire. The Gold Coast runs east of here up to and including the Volta River. The Bight of Benin covers the Volta River in Ghana to the Nun River in the Niger delta of Nigeria, and the Bight of Biafra, east of the Nun River to Cape Lopez in modern Gabon, inclusive. West-central Africa is defined as the rest of the western coast of the continent south of this point, and Southeast Africa anywhere east of the Cape of Good Hope [35]. The Windward coast was combined with Sierra Leone due to poor representation in the mtDNA literature, a practice often found in the historic literature as well [23]. Ethnic groups contributed in each group are summarized in Additional file 5: Table S5.

### mtDNA analyses and haplogroup determination

After exclusion criteria, analyses were preformed on 400 Jamaican individuals from around the island. Buccal swabs were collected from all subjects and stored in cell lysis solution (Medical Packaging Corporation, Camarillo, CA). Total DNA was then extracted using the Qiagen buccal cell spin protocol (Qiagen Ltd., Crawley, UK). The HVS-1 was then amplified using PCR. A reverse primer was used to generate all sequences (for details see [36]). A forward primer was also used to read the sequence prior to the polycytosine tract between base pairs 16184-16193 according to the revised mtDNA reference sequence (NCBI Reference Sequence: NC_012920.1). Sequencing product was then separated by 5% denaturing Long Ranger gel and detected using an Applied Biosystems 377 DNA sequencer (FMC Bio-Products, Rockland, ME, USA). Chromatograms were later read and converted to text using Chromas Lite (Chromas Lite, Technelysium, Queensland, Australia), then manually aligned to the reference sequence (rCRS) using Bioedit [37]. Differences from the rCRS were recorded, controlling for the length polymorphism found between base pairs 16184-16193, and haplogrouped according to the comprehensive full mtDNA genome phylogenetic tree Phylotree Build 11 (7 Feb 2011) [38] and the comprehensive mtDNA database published alongside the Genographic Project [22] at a comparable resolution to that in the literature [12]. In order to monitor sequencing errors that could arise from sequencing, phylogenetic inconsistencies were monitored using phylogenetic tools [39-44]. When observing some phylogenetic inconsistency, sequencing electropherograms were rechecked; DNA sample was re-extracted or re-sequenced when persisting some doubt regarding the variant in question. Additional coding region SNP genotyping was performed on ambiguous sequences to aid in resolution.

### Data analysis

Admixture coefficients based on haplogroup profile distributions were estimated using a Markov-Chain Monte Carlo posterior sampling method assuming a multinomial distribution for the mtDNA haplogroup profiles, a method best explained in detail elsewhere [8]. Additionally, admixture coefficients were also calculated based on shared haplotype frequencies and explored in a Bayesian framework, best explained in detail elsewhere [45]. An exact test of population differentiation [46] was performed on haplogroup profile frequencies between each African coast to investigate the discrete nature of each group in light of any effect population migrations or the inter-African slave trade have may played on the clustering of populations. All haplotypes not found in Sub-Saharan Africa were excluded from the Jamaican sample for the admixture analyses in order to focus the analysis only on African groups and accommodate the historical embarkation data from the continent. Additional analyses were preformed excluding more marginal groups with debatable influence on the slave trade, including the pygmy populations of equatorial Africa and populations from the Sahel, a region geographically defined here as 11.25-18.75°N, 16.875°W-35.625°E [47].

In order to investigate the internal diversity of the studied regions' mtDNA pools, the following diversity indices were calculated: number of different haplotypes (k), number of polymorphic sites (S), mean number of pairwise differences (θπ) [48]. Demographic properties of the population samples were assessed against Tajimas's D neutrality statistic, a parameter that investigates the nucleotide sequence diversity with respect to a mutation parameter calculated using the number of segregating sites [49]. Additionally, a mismatch analysis with 1000 bootstrap replicates was preformed to validate the unimodal stepwise expansion model based on the pairwise distribution using an index of raggedness (RI) [50] and sum of squared deviations (SSD) [51]. All parameters were calculated using Arlequin 3.5 [52].

## Abbreviations

mtDNA: mitochondrial DNA.

## Authors' contributions

MLD conceived the study, performed the genetic laboratory analysis, data analysis, and drafted the manuscript. AS aided in the data analysis, interpretation and presentation. SPN assisted in the social science interpretation and validation of historical content. VAM helped in the design, conception and analysis. EYstAM aided in sample collection and help in the study design. YPP was the principal sample collector and aided in the study design. All authors reviewed and commented on the manuscript during its drafting and approved the final version.

## Supplementary Material

Additional file 1 **Table S1**. HVS-I sequences of the Jamaican individuals analyzed in the present study. Click here for file

Additional file 2 **Table S2**. Haplogroup frequencies of Jamaica, different African regions and different iterations considered and analyzed in the present study. Click here for file

Additional file 3 **Table S3**. Haplotypes shared (relative frequencies) between Jamaica and the different African regions analyzed in the present study. Click here for file

Additional file 4 **Table S4**. Number of shared haplotypes between the different regions considered in the present study. Numbers in the diagonal are the number of different haplotypes per region. In brackets is the proportion of the Jamaican haplotypes that are shared with each of the African regions considered. Click here for file

Additional file 5 **Table S5**. Mitochondrial DNA HVS-I sequences included in this study [15,53-80]. Click here for file
