# Supplementary material for: Interdisciplinary approach to the demography of Jamaica
Source: BMC Evol Biol. 2012 Feb 23;12:24. doi: 10.1186/1471-2148-12-24 (PMC3299582; doi:10.1186/1471-2148-12-24)
Supplement: Additional file 5 — Table S5. Mitochondrial DNA HVS-I sequences included in this study [15,53-80]. [file 1471-2148-12-24-S5.DOC]

| **Supplementary table 5. Mitochondrial DNA HVS-I sequences included in this study** | | | | | | |
| --- | --- | --- | --- | --- | --- | --- |
|  |  |  |  |  |  |  |
|  | **ethnicity** | **country** | **n** | **Sahel** | **Pygmie** | **Reference** |
| **Senegambia** | |  |  |  |  |  |
|  | Bijago | Guinea-Bissau | 22 | no | no | [53] |
|  | Multiple groups | Guinea-Bissau | 17 | no | no | [53] |
|  | Wolof | Senegal | 131 | yes | no | [54]; [55]; Ely et al. unpublished |
|  | Mandenka | Senegal | 110 | yes | no | [56] |
|  | Malinke | Senegal | 93 | yes | no | [57]; Ely et al. unpublished |
|  | Balanta | Guinea-Bissau | 70 | yes | no | [53]; Ely et al. unpublished |
|  | Joola | Senegal | 49 | yes | no | Ely et al. unpublished |
|  | Fula | Guinea-Bissau | 38 | yes | no | [53] |
|  | Peul | Senegal | 32 | yes | no | [57]; [55]; Ely et al. unpublished |
|  | Serer | Senegal | 30 | yes | no | [54]; Ely et al. unpublished |
|  | Manjaco | Guinea-Bissau | 27 | yes | no | [53] |
|  | Malu | Guinea-Bissau | 26 | yes | no | [53] |
|  | Papel | Guinea-Bissau | 24 | yes | no | [53]; Ely et al. unpublished |
|  | Tuareg | Senegambia | 24 | yes | no | [56]; [57] |
|  | Mancanha | Guinea-Bissau | 20 | yes | no | [53]; Ely et al. unpublished |
|  | Beafada | Guinea-Bissau | 19 | yes | no | [53] |
|  | Fula-Preto | Guinea-Bissau | 19 | yes | no | [53] |
|  | Futa-Fula | Guinea-Bissau | 19 | yes | no | [53] |
|  | Djola | Guinea-Bissau | 18 | yes | no | [53] |
|  | Mansonca | Guinea-Bissau | 18 | yes | no | [53] |
|  | Tukulor | Senegal | 14 | yes | no | [54]; [55] |
|  | Diola | Senegal | 11 | yes | no | [54]; [55] |
|  | Multiple groups | Senegal | 16 | yes | no | Ely et al. unpublished |
|  | Multiple groups | Senegal | 8 | yes | no | [54] |
|  | Soninke | Senegal | 8 | yes | no | [54]; [57] |
|  | Multiple groups | Guinea-Bissau | 15 | yes | no | [53] |
|  | Multiple groups | Senegal | 7 | yes | no | [55] |
|  | Lebou | Senegal | 4 | yes | no | [55]; Ely et al. unpublished |
|  | Maure | Senegal | 3 | yes | no | [55]; Ely et al. unpublished |
| **Total** |  |  | **892** | **39** | **892** |  |
|  |  |  |  |  |  |  |
| **Sierra Leone** | |  |  |  |  |  |
|  | Ahizi | Ivory Coast | 128 | no | no | [14] |
|  | Temne | Sierra Leone | 121 | no | no | [58] |
|  | Sierra Leone | Sierra Leone | 109 | no | no | [18] |
|  | Bambara | Mali | 79 | no | no | [57]; [55]; Ely et al. unpublished |
|  | Limba | Sierra Leone | 67 | no | no | [58] |
|  | Yacouba | Ivory Coast | 62 | no | no | [14] |
|  | Mende | Sierra Leone | 59 | no | no | [58] |
|  | Loko | Sierra Leone | 29 | no | no | [58] |
|  | Bamana | Mali | 3 | no | no | Ely et al. unpublished |
|  | Baoulé | Ivory Coast | 1 | no | no | [14] |
|  | Kru | Liberia | 1 | no | no | Ely et al. unpublished |
|  | Fulani Banfora | Burkina Faso | 59 | yes | no | [59] |
|  | Mandinga | Guinea-Bissau | 30 | yes | no | [53] |
|  | Manjak | Guinea-Bissau | 22 | yes | no | Ely et al. unpublished |
|  | Senoufo | Mali | 12 | yes | no | [57]; Ely et al. unpublished |
|  | Mandingo | Guinea-Bissau | 11 | yes | no | Ely et al. unpublished |
|  | Bobo | Mali | 8 | yes | no | [57]; Ely et al. unpublished |
|  | Multiple groups | Mali | 8 | yes | no | Ely et al. unpublished |
|  | Dogon | Mali | 7 | yes | no | [57]; Ely et al. unpublished |
|  | Sonrhai | Mali | 6 | yes | no | [57] |
|  | Burkinabè | Burkina Faso | 1 | yes | no | [14] |
| **Total** |  |  | **823** | **659** | **823** |  |
|  |  |  |  |  |  |  |
| **Gold Coast** | |  |  |  |  |  |
|  | Akan | Ghana | 275 | no | no | [19], Ely et al. unpublished |
|  | Ewe | Ghana | 115 | no | no | [19], Ely et al. unpublished |
|  | Gaa-Adangbe | Ghana | 77 | no | no | Ely et al. unpublished |
|  | Multiple groups | Ghana | 24 | no | no | Ely et al. unpublished |
|  | Songhai | Burkina Faso | 10 | yes | no | [56] |
|  | Mossi | Burkina Faso | 2 | yes | no | Ely et al. unpublished |
|  | Songhoy | Burkina Faso | 2 | yes | no | Ely et al. unpublished |
| **Total** |  |  | **505** | **491** | **505** |  |
|  |  |  |  |  |  |  |
| **Bight of Benin** | |  |  |  |  |  |
|  | Yoruba | Nigeria | 156 | no | no | [56]; [14]; Ely et al. unpublished |
|  | Fon | Benin | 79 | no | no | [14] |
|  | Aïzo | Benin | 11 | no | no | [14] |
|  | Goun | Benin | 11 | no | no | [14] |
|  | Multiple groups | Benin | 40 | no | no | [14] |
|  | Hausa | Nigeria/Niger | 68 | yes | no | [56]; [14]; Ely et al. unpublished |
|  | Fulani Tindangou | Burkina Faso | 54 | yes | no | [59] |
|  | Multiple groups | Benin | 2 | yes | no | [14] |
| **Total** |  |  | **421** | **297** | **421** |  |
|  |  |  |  |  |  |  |
| **Bight of Biafra** | |  |  |  |  |  |
|  | Ibibio | Nigeria | 509 | no | no | [19] |
|  | Igbo | Nigeria | 293 | no | no | [19] |
|  | Efik | Nigeria | 145 | no | no | [19] |
|  | Ejagham | Nigeria | 133 | no | no | [19] |
|  | Ngumba | Cameroon / Equitorial Guinea | 132 | no | no | [60]; [61] |
|  | Fang | Cameroon | 116 | no | no | [62]; [61] |
|  | Aghem | Cameroon | 115 | no | no | [19] |
|  | Bamun | Cameroon | 109 | no | no | [19]; Ely et al. unpublished |
|  | Annang | Nigeria | 107 | no | no | [19] |
|  | Oron | South East Nigeria | 98 | no | no | [19] |
|  | Fulbe | Niger / Cameroon | 94 | no | no | [56]; [63] |
|  | Ewondo | Cameroon | 81 | no | no | [63]; [61], Ely et al. unpublished |
|  | Fali | Cameroon | 81 | no | no | [63]; [64] |
|  | Bamileke | Cameroon | 53 | no | no | [63]; Ely et al. unpublished |
|  | Bakaka | Cameroon | 50 | no | no | [63] |
|  | Benga | Gabon | 50 | no | no | [61] |
|  | Bassa | Cameroon | 46 | no | no | [63] |
|  | Bubi | Equitorial Guinea | 45 | no | no | [65] |
|  | Podoko | Cameroon | 43 | no | no | [63]; Ely et al. unpublished |
|  | Mandara | Cameroon | 37 | no | no | [63] |
|  | Uldeme | Cameroon | 28 | no | no | [63] |
|  | Tupuri | Cameroon | 25 | no | no | [63] |
|  | Daba | Cameroon | 20 | no | no | [63] |
|  | Tali | Cameroon | 20 | no | no | [63] |
|  | Multiple groups | Cameroon | 34 | no | no | Ely et al. unpublished |
|  | Baka | Cameroon | 177 | no | yes | [60]; [61] |
|  | Bakola | Cameroon | 137 | no | yes | [60]; [61] |
|  | Biaka | Central Africa Republic | 73 | no | yes | [61]; [56] |
|  | Tikar | Cameroon | 69 | no | yes | [61]; [19] |
|  | W.Mbenzele | Cameroon | 57 | no | yes | [66] |
|  | Bakoya | Gabon | 31 | no | yes | [61] |
|  | Fulani Bongor | Chad | 105 | yes | no | [59]; [64] |
|  | Chadic-Cameroon | Cameroon | 104 | yes | no | [67] |
|  | Fulani Tcheboua | Cameroon | 86 | yes | no | [59]; [64] |
|  | Kotoko | Cameroon | 56 | yes | no | [64] |
|  | Kanembu | Lake Chad | 50 | yes | no | [64] |
|  | Kanuri | Nigeria/Cameroon/Sudan/Niger | 49 | yes | no | [56]; [64]; Ely et al. unpublished |
|  | Arabs Shuwa | Lake Chad | 38 | yes | no | [64] |
|  | Mafa | Lake Chad | 32 | yes | no | [64] |
|  | Masa | Lake Chad | 32 | yes | no | [64] |
|  | Buduma | Lake Chad | 30 | yes | no | [64] |
|  | Arabs Chad | Lake Chad | 27 | yes | no | [64] |
|  | Hide | Lake Chad | 23 | yes | no | [64] |
|  | Arab Choa | Cameroon | 1 | yes | no | Ely et al. unpublished |
| **Total** |  |  | **3641** | **3008** | **3097** |  |
|  |  |  |  |  |  |  |
| **West-central Africa** | |  |  |  |  |  |
|  | Nyaneka-Nkhumbi | Angola | 153 | no | no | [68] |
|  | Cabinda | Angola | 110 | no | no | [69] |
|  | Ovimbundu | Angola | 92 | no | no | [68] |
|  | Mitsogo | Gabon | 64 | no | no | [61] |
|  | Nzebi | Gabon | 63 | no | no | [61] |
|  | Kota | Gabon | 56 | no | no | [61] |
|  | Ateke | Gabon | 54 | no | no | [61] |
|  | Kuvale | Angola | 54 | no | no | [68] |
|  | Punu | Gabon | 52 | no | no | [61] |
|  | Galoa | Gabon | 51 | no | no | [61] |
|  | Shake | Gabon | 51 | no | no | [61] |
|  | Bateke | Popular Repulic of Congo | 50 | no | no | [60] |
|  | Akele | Gabon | 48 | no | no | [61] |
|  | Duma | Gabon | 47 | no | no | [61] |
|  | Obamba | Gabon | 47 | no | no | [61] |
|  | Angoloan Others | Angola | 45 | no | no | [68] |
|  | Makina | Gabon | 45 | no | no | [61] |
|  | Mbundu | Angola | 43 | no | no | [70] |
|  | Eshira | Gabon | 40 | no | no | [61] |
|  | Ndumu | Gabon | 39 | no | no | [61] |
|  | Eviya | Gabon | 38 | no | no | [61] |
|  | Sanga | Central African Republic | 30 | no | no | [60] |
|  | Ganguela | Angola | 21 | no | no | [68] |
|  | Orungu | Gabon | 20 | no | no | [61] |
|  | Bakongo | Angola | 1 | no | no | [70] |
|  | Babongo | Gabon | 45 | no | yes | [61] |
|  | Babinga | Democratic Repulic of Congo | 44 | no | yes | [60] |
| **Total** |  |  | **1403** | **1403** | **1314** |  |
|  |  |  |  |  |  |  |
| **South-east Africa** | |  |  |  |  |  |
|  | Bantu-speaking | Mozambique | 109 | no | no | [71] |
|  | !Kung | Namibia | 96 | no | no | [72]; Ely et al. unpublished; [56] |
|  | Shona | Zimbabwe | 76 | no | no | [73]; [74] |
|  | Antandroy | Madagascar | 59 | no | no | [75] |
|  | Antanosy | Madagascar | 54 | no | no | [75] |
|  | Khwe | Namibia | 31 | no | no | [72] |
|  | Chopi | Mozambique | 27 | no | no | [73] |
|  | Ronga | Mozambique | 22 | no | no | [73] |
|  | Shangaan | Mozambique | 22 | no | no | [73] |
|  | Sena | Mozambique | 21 | no | no | [73] |
|  | Sukuma | Tanzania | 21 | no | no | [76] |
|  | Chwabo | Mozambique | 20 | no | no | [73] |
|  | Lomwe | Mozambique | 20 | no | no | [73] |
|  | Makhuwa | Mozambique | 20 | no | no | [73] |
|  | Nyanja | Mozambique | 20 | no | no | [73] |
|  | Nyungwe | Mozambique | 20 | no | no | [73] |
|  | Tonga | Mozambique | 20 | no | no | [73] |
|  | Herero | Botswana | 19 | no | no | Ely et al. unpublished |
|  | Makonde | Mozambique | 19 | no | no | [73] |
|  | Ndau | Mozambique | 19 | no | no | [73] |
|  | Tswa | Mozambique | 19 | no | no | [73] |
|  | Antaisaka | Madagascar | 11 | no | no | [75] |
|  | Nguni | Mozambique | 11 | no | no | [73] |
|  | Yao | Mozambique | 10 | no | no | [73] |
|  | Merina | Madagascar | 9 | no | no | [75] |
| **Total** |  |  | **775** | **775** | **775** |  |
|  |  |  |  |  |  |  |
| **East Africa** | |  |  |  |  |  |
|  | Amhara | Ethiopia | 127 | no | no | [77] |
|  | Kenya | Kenya | 100 | no | no | [78] |
|  | Luhya | Kenya | 94 | no | no | Ely et al. unpublished |
|  | Hadza | Tanzania | 66 | no | no | [76]; Ely et al. unpublished |
|  | Oromo | Ethiopia | 51 | no | no | [79]; [80] |
|  | Dinka | Sudan | 47 | no | no | [81] |
|  | Tigrai | Ethiopia | 45 | no | no | [80] |
|  | Hutu | Rwanda | 42 | no | no | [74] |
|  | Turkana | Kenya | 37 | no | no | [56] |
|  | Somali | Somalia | 27 | no | no | [56] |
|  | Kikuyu | Kenya | 24 | no | no | [56] |
|  | Gurage | Ethiopia | 21 | no | no | [80] |
|  | Datoga | Tanzania | 18 | no | no | [76] |
|  | Afar | Ethiopia | 16 | no | no | [80] |
|  | Iraqw | Tanzania | 12 | no | no | [76] |
|  | Nuer | Sudan | 11 | no | no | [81] |
|  | Eritrean | Eritrea | 8 | no | no | [80] |
|  | Shilluk | Sudan | 7 | no | no | [81] |
|  | Mbuti | Democratic Republic of Congo | 52 | no | yes | [56]; [61] |
| **Total** |  |  | **805** | **805** | **753** |  |
